# Comprehensive Chemical Characterization of Chia (*Salvia hispanica* L.) Seed Oil with a Focus on Minor Lipid Components

**DOI:** 10.3390/foods12010023

**Published:** 2022-12-21

**Authors:** Marianna Oteri, Giovanni Bartolomeo, Francesca Rigano, Juan Aspromonte, Emanuela Trovato, Giorgia Purcaro, Paola Dugo, Luigi Mondello, Marco Beccaria

**Affiliations:** 1 Department of Veterinary Sciences, Section of Animal Production, University of Messina, I-98168 Messina, Italy; 2 Science4Life S.r.l., an Academic Spin-Off of University of Messina, I-98168 Messina, Italy; 3 Department of Chemical, Biological, Pharmaceutical, and Environmental Sciences, University of Messina, I-98168 Messina, Italy; 4 Laboratorio de Investigación y Desarrollo de Métodos Analíticos, LIDMA, Facultad de Ciencias Exactas, Universidad Nacional de La Plata, CIC-PBA, CONICET, Calle 47 esq. 115, La Plata 1900, Argentina; 5 Gembloux Agro-Bio Tech, University of Liège, Passage des Déportés 2, 5030 Gembloux, Belgium; 6 Chromaleont s.r.l., c/o Department of Chemical, Biological, Pharmaceutical and Environmental Sciences, University of Messina, I-98168 Messina, Italy; 7 Unit of Food Science and Nutrition, Department of Medicine, University Campus Bio-Medico of Rome, I-00128 Rome, Italy; 8 Department of Chemical, Pharmaceutical, and Agricultural Sciences (DOCPAS), Via Luigi Borsari 46, University of Ferrara, 44121 Ferrara, Italy

**Keywords:** chia seed oil, essential fatty acids, lipidomics, chromatographic techniques, NanoLC-EI-MS, mass spectrometry, minor lipid compounds, ω3 fatty acids, nutritional indices

## Abstract

A comprehensive chemical characterization of different lipid components, namely fatty acid composition after derivatization in fatty acid methyl esters (FAMEs), triacylglycerols (TAGs), phospholipids (PLs), free fatty acids (FFAs), sterols, carotenoids, tocopherols, and polyphenols in Chia seed oil, obtained by Soxhlet extraction, was reported. Reversed phase liquid chromatography (RP-LC) coupled to UV and mass spectrometry (MS) detectors was employed for carotenoids, polyphenols, and TAGs determination; normal phase-LC in combination with fluorescence detector (FLD) was used for tocopherols analysis; PL and FFA fractions were investigated after a rapid solid phase extraction followed by RP-LC-MS and NanoLC coupled to electron ionization (EI) MS, respectively. Furthermore, gas chromatography (GC)-flame ionization (FID) and MS detectors were used for FAMEs and sterols analysis. Results demonstrated a significant content of bioactive compounds, such as the antioxidant tocopherols (22.88 µg mL^−1^), and a very high content of essential fatty acids (81.39%), namely α-linolenic (62.16%) and linoleic (19.23%) acids. In addition, for the best of authors knowledge, FFA profile, as well as some carotenoid classes has been elucidated for the first time. The importance of free fatty acids in vegetable matrices is related to the fact that they can be readily involved in metabolic processes or biosynthetic pathways of the plant itself. For a fast and reliable determination of this chemical class, a very innovative and sensitive NanoLC-EI-MS analytical determination was applied.

## 1. Introduction

Chia (*Salvia hispanica* L.) is an herbaceous plant, belonging to the *Lamiaceae* family, cultivated annually and native of the region that goes from southern Mexico to northern Guatemala [1,2]. Nowadays, chia is mainly cultivated in Mexico, Paraguay, Peru, Bolivia, Argentina, Colombia, Ecuador, Australia, and Guatemala as a seed crop and used as food, animal feed, and a source of pharmaceuticals and nutraceuticals, due to its content in bioactive molecules [3,4,5,6].

From a nutritional point of view, chia seeds contain 16% of proteins, 30% of total lipids, 42% of carbohydrates, of which 34% is dietary fiber. As for minor constituents, seeds have a favorable mineral and vitamin composition and contain micronutrients such as polyphenols, carotenoids, and anthocyanins [7]. In fact, chia seeds are recommended as a well-balanced food product. Indeed, their protein content is higher than linseed, traditional cereals (wheat, corn, rice, oats, and barley), and pseudo-cereals, such as amaranth and quinoa [2,8,9]. Moreover, they also present a high oil content (ranged from 20% to 40%). Therefore, the production of chia oil represents one of the main uses of chia seeds.

The difference in terms of chemical composition depends on several factors, encompassing the combination of the cultivation environment (i.e., pedoclimatic conditions) and the genotype [10,11]. Nevertheless, when used for oil production, the extraction process will also strongly influence the yield and composition of the chia seed oil, eventually affecting its nutritional value. For instance, Ixtaina et al. (2011) [12] reported that oil yields obtained by pressing are much lower than those obtained by solvent extraction.

Regarding the chemical composition of chia oil, previous studies [13,14] pointed out that chia is also known as one of the best plant sources of omega-3 (ω-3) fatty acids (FAs), particularly of α-linolenic acid (ALA), leading to an impressive content of polyunsaturated fatty acids (PUFA) (ALA, up to 68% and ω-6 linoleic acid, 20%) [10,11,12,15,16,17,18,19], compared to other known plant sources [1,9,10,20,21]. 

ALA and ω-6 linoleic acid are both essential fatty acids, and thus not synthesizable and required by the human body for optimal health [4,22]. ALA is the precursor of long-chain ω-3 FAs, which are important for the prevention and treatment of cardiovascular diseases [23], since they promote the synthesis of anti-inflammatory molecules. Moreover, they positively contribute to the development and maintenance of the brain and nervous system [5], as well as to the control of blood glucose levels [2,22,24], and enhance the autoimmune defenses [23].

Furthermore, the presence of minor compounds, playing different beneficial roles for human and animal health, was also investigated in chia seed oil. Tocopherols, polyphenols, carotenoids, and phospholipids (PLs) containing PUFAs were related to the prevention of cancerous, cardiovascular, and inflammatory diseases thanks to their antioxidant activity [3,12,25], whereas chia phytosterols showed a hypocholesterolemic action through the reduction of cholesterols levels in human blood.

Although both chia seeds and chia oil have been extensively studied to date [3,4,12,14,26,27,28], a comprehensive chemical evaluation of intact lipids, including minor lipid compounds, has, to the best of our knowledge, not yet been reported. According to European Commission and European Food Safety Authority (EFSA), chia seeds are included in the category “novel food” [29]. This has brought a new interest in the study of the components present in chia seed-related products such as chia seed oil.

Within this context, the elucidation of lipids in their native form rather than the evaluation of the total fatty acid composition is needed to deeply investigate the nutritional properties of such a functional food. In fact, each lipid class can be differently involved in metabolic processes. The aim of the present work was to achieve a comprehensive characterization of intact lipids in chia oil, obtained from *Salvia hispanica* L. seeds by solvent extraction, with a focus on minor lipid components such as free fatty acids (FFAs) and PLs, using high-performance chromatographic techniques. Conventional chromatographic approaches were used in most cases, such as high-performance liquid chromatography (HPLC) and gas chromatography (GC). The first was employed for the determination of non-volatile components (TAGs, PLs, polyphenols, carotenoids, and tocopherols), while the second was used for the determination of the more volatile compounds (FAs and sterols). The latter two classes (FAs and sterols) were converted into methyl esters and trimethylsilyl derivatives, respectively, to further reduce their polarity and boiling points, making them more GC-amenable. It should be noted that the total FA determination does not consider the lipid structure in which they are bound, since all the saponifiable lipids containing FA molecules are converted into fatty acid methyl esters (FAMEs). 

Hence, FFAs were isolated and analyzed through a previously validated innovative NanoLC method [30]. This method has the great advantage of injecting FFAs in their native form without the need for any derivatization procedure, reducing the chances of a poor method accuracy due to the formation of artifacts. Moreover, the miniaturization of the LC system enables its direct hyphenation with electron ionization mass spectrometry (EI-MS), a typical GC detector. The highly reproducible and informative EI-MS spectra allows for a univocal and reliable identification of single FFAs. The developed NanoLC-EI-MS system represents an innovative prototype instrument that brings the benefits of GC-MS to LC-amenable compounds that can be fast and automatically identified, thanks to the commercially available EI-MS spectral libraries [31]. 

To date, no work concerning the characterization of FFAs in chia seed oil has been reported. 

## 2. Materials and Methods

### 2.1. Chemical and Reagents

*n*-hexane (Hex), methanol (MeOH), 2-propanol (IPA), acetonitrile (ACN), water (H_2_O) (GC and LC-MS grade solvents), potassium hydroxide (KOH), chloroform, diethyl ether, acetic acid, ammonium formate, formic acid, N,N-dimethyl-formamide (DMF), petroleum ether, methyl tert-butyl ether (MTBE), phenolphthalein, sodium sulphate, 2,7-dichlorofluorescein and BSTFA (N,O-Bis(trimethylsilyl)trifluoroacetamide), were purchased from Merck Life Science (Darmstadt, Germany).

All the analytical standards for GC and LC analyses (dihydrocholesterol, C4-C24 FAMEs Supelco mix, α-tocopherol, γ-tocopherol, δ-tocopherol and lutein) were provided by Merck Life Science (Merck KGaA, Darmstadt, Germany). The 500 mg/6 mL Bond Elut NH_2_ solid-phase extraction (SPE) cartridge was purchased from Agilent Technologies (Santa Clara, CA, USA).

### 2.2. Sample and Sample Preparation

Chia (*Salvia hispanica* L.) seeds were provided by a local pharmacy. The seeds were pounded and oil extraction was carried out using a Soxhlet apparatus with *n*-hexane, according to IUPAC standardized method [32].

### 2.3. Fatty Acid Methyl Ester Analysis 

Complex lipids, including acylglycerols, phospholipids, waxes and sterols esters, were transformed in FAMEs by adding 1 mL of Hex and 1 mL of 2 N methanolic solution of potassium hydroxide to 100 µL of the oil, shaking for 15 s, and incubating the mixture at room temperature for 5 min. The upper layer, containing the FAMEs, was analyzed by means of a GC- flame ionization detector (FID) and a GC-MS system (both from Shimadzu, Kyoto, Japan), for quantitative and qualitative purposes, respectively. The procedure was carried out in triplicate.

The GC-FID system consisted of a GC-2010 oven equipped with, an AOC-20i autosampler and an FID detector. The GC-MS instrument consisted of a GCMS-QP2010 oven equipped with an AOC-20i autosampler and a quadrupole q-MS detector provided with an electron ionization (EI) source (70 eV ionization energy).

Chromatographic separation of FAMEs was achieved on a Supelcowax-10 (Merck KGaA, Darmstadt, Germany) 30 m × 0.25 mm, (L × I.D.), 0.25 μm film thickness capillary column operated under a programmed temperature (50–280 °C at 3.0 °C/min), using He as carrier gas at a constant linear velocity of 30 cm/s (inlet pressure of 99.4 and 26.6 kPa at the GC-FID and GC-MS systems, respectively). The injector temperature was set at 280 the °C, the injection volume was 0.1 μL, and the split ratio was 10:1 and 200:1 for GC-FID and GC-MS analyses, respectively.

GC-FID parameters were set as follows: detector temperature, 280 °C; H_2_ flow rate, 50 mL/min; air flow rate, 400 mL/min; make-up gas (N_2_) flow rate, 50 mL/min; sampling frequency, 80 ms; filter time constant, 200 ms. 

The following MS parameters were employed: mass range 40–400 amu; scan speed: 2000 amu/s; ion source temperature 200 °C; interface temperature: 250 °C.

The GC-MS Solution software (version 4.50, Shimadzu Kyoto, Japan) was used for GC-MS data collection and handling for identification purposes through an automatic search by loading Lipids GC-MS Library Version 1.0 (Shimadzu, Kyoto, Japan) with the embedded Linear Retention Index (LRI) library. LRI values were calculated for all target compounds by injecting a reference standard mixture of even carbon number FAMEs C4-C24 under the same analytical conditions as the samples.

LabSolution software (version 5.91, Shimadzu Kyoto, Japan) was employed for GC-FID data collection and handling for quantitative purposes.

Particularly, relative quantification was carried out by integrating each peak and calculating area percentages, considering a quite identical FID response for the identified FAMEs.

Atherogenic (AI) and thrombogenic (TI) nutritional indices and hypocholesterolemic/hypercholesterolemic ratio (h/H) were calculated from identified FAs according to the equations reported by Chen and Liu [33], as reported below (Equations (1) to (3)):

AI = [C12:0 + (4 × C14:0) + C16:0]/ƩUFA(1)

TI = (C14:0 + C16:0 + C18:0)/[(0.5 × ƩMUFA) + (0.5 × Ʃn6-PUFA) + (3 × Ʃn3-PUFA) + (n3/n6)],(2)

h/H = (cis 18:1 + ƩPUFA)/(C12:0 + C14:0 + C16:0) (3)

### 2.4. Free Fatty Acid Analysis 

The FFA fraction was isolated by SPE [34]. Briefly, the NH_2_ SPE cartridge was preconditioned with Hex, then about 60 mg of oil was loaded. Neutral lipids (acylglycerols, waxes, carotenoids, sterols, and sterol esters) were first eluted with 4 mL of chloroform/IPA (2:1 *v/v*) and discarded. The following fraction, containing FFAs, was collected with 8 mL of diethyl ether/acetic acid (98:2, *v/v*). Finally, PLs were eluted with 4 mL of MeOH and analyzed later (Section 2.6). FFAs were dried under a stream of cold nitrogen and reconstituted in ACN/IPA (1:1, *v/v*) prior to NanoLC-EI-MS analysis. 

Analyses were performed using a Nano-prominence LC system coupled to a GCMS-QP2010 Ultra system (Shimadzu, Kyoto, Japan), using a previously reported configuration [35]. Separation of FFAs was achieved on a lab-made packed Nano-column, ReproSyl-Pur C18 (250 × 0.075 mm, L. × I.D., 3 μm *dp*), kindly provided by Prof. Gasparrini (Sapienza University of Rome). Water (solvent A) and ACN/IPA (90:10, *v/v*) (solvent B), both acidified with 0.1% acetic acid, were employed as mobile phases using the following gradient mode: 0–0.5 min, 0–50 % B; 0.5–30 min, 50–100 %B; held for 30 min, then to 0 % B in 5 min. The flow rate was 150 nL/min, while the injection volume was 30 nL. 

The NanoLC-EI-MS oven and interface were kept at 40 °C, while the ion source temperature was set at 300 °C. Analyses were conducted in the full scan mode (mass range of 80–400 *m/z*) and single ion monitoring (SIM) mode by using a 1.3 Hz acquisition rate. The complete spectra across the full mass range were used for identification, while the ions specified in SIM mode were used for the relative quantification. C12:0 was used as internal standard (IS). The choice of the *m/z* values was a very delicate analytical issue since it is necessary that the selected ion provide a good signal for both the analytes and the IS. Namely, saturated and unsaturated FFAs were determined by monitoring respectively the ions at 73 *m/z* and 69 *m/z*. These ions were selected as a compromise between the ion abundance in the relative spectra reported in commercially available libraries and their poor signal in the experimental baseline, in order to achieve a high signal-to-noise ratio. The ionization energy was 70 eV, while the detector voltage was 0.92 kV. LC conditions were controlled by Nano-Assist software Version 1.00 (Shimadzu. Kyoto. Japan), while MS parameters were set by GCMS Solution v. 2.70 (Shimadzu, Kyoto, Japan), also used for data acquisition and processing. 

Analyte identification was achieved by comparison between the experimental mass spectra and spectra from commercially available EI-MS databases, namely the W11N17 library (Wiley11-NIST17, Wiley, Hoboken, NJ, USA). Semi-quantitative analyses were performed using the response factor (RF) approach, after calculating the RF for each FFA commercial standard relative to the IS (C12:0), as previously validated [30].

### 2.5. Triacylglycerol Analysis 

For the analysis of TAGs, 10 mg of chia oil was dissolved in 1 mL of ACN/IPA (50/50 *v/v*). Non-Aqueous (NA)-reversed-phase (RP)-HPLC analysis of TAGs was carried out on a Shimadzu HPLC system (Kyoto, Japan) equipped with a CBM-20 A controller, two LC-20AD pumps, a DGU-20A5 degasser, a SIL-20AC autosampler, and an LCMS-2020 mass spectrometer (Shimadzu, Kyoto, Japan) equipped with an atmospheric pressure chemical ionization (APCI) interface (Shimadzu, Kyoto, Japan), by using a previously developed chromatographic method [36].

A Merck (Merck KGaA, Darmstadt, Germany) Ascentis Express C18 column (150 × 4.6 mm, L. × I.D., 2.7 µm *dp*) was used for the chromatographic separation. Mobile phases consisted of ACN (A) and IPA (B) at a flow rate of 1 mL/min in the following linear gradient elution mode: 0–50 min, 0–70% B; 50–55 min, 70% B; 56 min 0% B. The injection volume was 2 µL and analyses were run at room temperature.

The following APCI-MS parameters were applied: APCI was set in positive mode; mass spectral range, 250–1200 *m/z*; event time, 0.5 sec; nebulizing gas (N_2_) flow, 4.0 L/min; APCI temp., 400 °C; Heat block temp., 230 °C; desolvation line (DL) temp., 250 °C; DL voltage, −34 V; probe voltage, +4.5 kV; Q-array voltage, 1.0 V, RF voltage, 90 V; detection gain, 1.05 kV. Data acquisition was processed through the LabSolution software (version 5.91, Shimadzu, Kyoto, Japan). Three replicates of analyses were acquired, and relative quantification was performed by using the APCI response factor [36,37].

### 2.6. Phospholipid Analysis 

The PL fraction, isolated by the SPE procedure described in Section 2.4, was dried by using a rotary evaporator at 30 °C (Hei-VAP Precision, Heidolph, Schwabach, Germany), re-dissolved in 1 mL of MeOH and analyzed by an RP-UHPLC-APCI^(+)^-qMS lipidomics methodology previously developed [38]. Briefly, an UHPLC RP C18 column (100 mm × 2.1 mm, L. × I.D., 1.9 μm *dp*, Merck KGaA, Darmstadt, Germany) was operated at a flow rate of 0.4 mL/min by using the following mobile phases A) H_2_O/ammonium formate 20 mM and B) IPA/ACN/H_2_O (60:36:4, *v/v/v*) and 0.1% formic acid under gradient elution mode (0–6 min, 80–100% B; 6–22 min, 100% B). The injection volume was 5 μL and the column oven was kept at 40 °C. 

APCI-MS acquisition was performed in full scan and SIM modes, in both positive and negative polarity, sequentially. Full scan chromatograms were obtained by scanning 350–1250 *m/z* in positive ionization mode and 150-1250 *m/z* in negative ionization mode, with an event time of 0.2 sec. Monitored ions in SIM were *m/z* 227.5, 241.5, 253.5, 255.5, 277.5, 279.5, 281.5, 283.5, 309.5 in negative mode (related to the main FAs present in chia oil). Interface, DL, and heat block temperatures were: 450 °C, 250 °C, and 200 °C, respectively; nebulizing gas (N_2_) flow was 3 L/min.

### 2.7. Sterol Analysis 

Sterols determination was performed using the official method UE N. 1348/2013 [39]. Briefly, 2.25 g of chia oil was subjected to a saponification reaction using 50 mL of methanolic solution of potassium hydroxide (2 N) with dihydrocholesterol (500 μL of a 0.2% *v/v* chloroform solution, evaporated to dryness prior of the addition of the oil) as IS, under reflux and magnetic stirring. After cooling, the reaction mixture was transferred into a separatory funnel and 50 mL of water and 50 mL of diethyl ether were added. The solution was shaken, and the upper organic phase was collected after separation of the two phases. The water phase was extracted 3 times with 50 mL of diethyl ether. The organic phases were pooled and washed with H_2_O until the soaps disappeared, by using a separatory funnel. Then, the diethyl ether phase was filtered using Na_2_SO_4_ and the solvent was evaporated using a rotary evaporator (Hei-VAP Precision, Heidolph, Schwabach, Germany).

The dried extract was dissolved in chloroform and the solution was placed on a glass TLC plate (Thin Layer Chromatography), with silica gel as the stationary phase, using 100 mL of petroleum ether/diethyl ether (6: 4, *v/v*) mixture as eluent. The TLC plate was placed in a developing chamber, then2,7-dichlorofluorescein was applied in order to detect the separation by means of a UV lamp. The sterol band was scratched, solubilized in 10 mL of chloroform, and transferred to a water bath for chloroform evaporation. Sterols were derivatized by adding 100 μL of BSTFA (1% TMCS) and 100 μL of pyridine and leaving the mixture to react for 30 min at room temperature. The sample was dried under a stream of nitrogen and dissolved in n-hexane prior to GC injection.

GC-FID analyses were performed using a GC DANI Master (Dani Instruments S.p.A., Milan, Italy), equipped with a split-splitless inlet (280 °C) and an FID detector (290 °C).

Sterols separation was performed on an SPB1 column (Merck KGaA, Darmstadt, Germany) (15 m × 0.2 mm, L. × I.D., 0.2 μm *dp*). The GC operating parameters were as follows: carrier gas (He) at a constant flow of 1 mL/min; programmed oven temperature: from 240 °C to 290 °C in 25 min at 2.0 °C/min; injection volume, 1.0 μL; split ratio, 1:100. Analyses of sterols were performed in triplicate.

### 2.8. Tocopherol Analysis 

Tocopherols analysis was carried out by using a Shimadzu HPLC system (Kyoto, Japan) equipped with a LC 10 AD Vp high-pressure isocratic pump, an SCL-10A Vp controller, and an RF-10 AXL fluorescence detector.

The chromatographic separation was performed using a micro-silica Ascentis SI column (250 × 1.0 mm, L. × I.D., 5 µm particle size, Merck KGaA, Darmstadt, Germany) at room temperature (25 °C), operated in isocratic mode with Hex/IPA (99:1, *v/v*) mobile phase and flow rate of 50 µL/min. 2 µL of chia oil (10 mg in 1 mL of Hex) were injected by using a rheodyne injector in triplicates. RF detector was programmed for excitation at wavelength λ = 290 nm and emission at λ = 330 nm. Data acquisition was performed using the LabSolution software (ver. 5.97, Shimadzu, Kyoto, Japan).

The method was validated following the EURACHEM guidelines for each component, namely α-tocopherol, γ-tocopherol, and δ-tocopherol [40]. Linearity was tested at 6 different concentrations for each analyte, performing five replicates per level. Regression lines were built using the least squares linear regression. The linearity and the goodness of the curves used were confirmed using Mandel’s fitting tests [41]. The significance of the intercept was established by running a *t*-test (significance level of 5%). Accuracy, in terms of trueness and precision, was assessed at two different levels. The Shapiro-Wilk test, to check the normality of the distribution, and the Dixon and Grubbs tests, to verify the presence of outliers, were performed before calculating precision in terms of coefficient of variation (CV%) and limit of repeatability (r). The limit of detection (LOD) and limit of quantification (LOQ) were calculated by performing 10 analyses of a blank sample and applying the following equations:

LOD: yd = μb + 2t × σb

LOQ: yq = μb + 10 × σb

where yd and yq is the signal at the LOD and LOQ, respectively, μb is the average signal of the blank sample, σb is the blank standard deviation and t is the constant of the t-Student distribution depending on the confidence level (95%) and degrees of freedom. Finally, LOD and LOQ values were obtained by plotting yd and yq in the calibration line.

### 2.9. Phenolic Compound Analysis 

Phenolic compounds were extracted from chia oil by using a slightly modified procedure already reported by Montedoro et al. (1992) [42]. Briefly, 3 g of oil were extracted with 6 mL of a MeOH/H_2_O (8:2, *v/v*) solution; the mixture was stirred and centrifuged for 10 min at 3000 rpm. Then, the oil phase was re-extracted with 6 mL of MeOH/H_2_O (8:2, *v/v*) mixture and the extraction procedure was repeated four more times, obtaining six methanolic fractions. All fractions were pulled and evaporated to dryness under vacuum at 30 °C by using a rotary evaporator (Hei-VAP Precision, Heidolph, Schwabach, Germany). The obtained residue was dissolved in 1 mL of ACN, treated with 1 mL of Hex to remove lipid components, and centrifuged for 10 min at 3000 rpm. The upper hexane phase was discarded, and this treatment was repeated two more times. Finally, the ACN solution containing polyphenols was dried using the rotary evaporator at 30 °C and the residue was dissolved in 500 µL of ACN/H_2_O (3:2, *v/v*) prior to the polyphenols analysis.

RP-LC analysis of polyphenols was performed by using the same instrumental setup and software described in Section 2.5, also equipped with a photodiode array detector (PDA) SPD-M20A (Shimadzu, Kyoto, Japan) directly connected to the LC column outlet and serially coupled with an ESI-MS source (Shimadzu, Kyoto, Japan). Chromatographic separation of analytes was achieved by using an Ascentis Express C18 column (150 × 4.6 mm, L. × I.D., 2.7 µm *dp,* Merck KGaA, Darmstadt, Germany), using a linear gradient mode (0–50 min 0–100% B, 50–55 min 100% B) of (A) H_2_O and (B) ACN mobile phases, both acidified with 0.1% formic acid. The flow rate was set at 1 mL/min (split to 0.4 mL/min prior to MS detection). The injection volume was 2 µL and the column was kept at room temperature.

PDA parameters were as follows: wavelength range was 210–400 nm, and the chromatograms were extracted at the maximum absorbance (280 nm). The sampling frequency was 6.25 Hz. ESI-MS acquisition parameters were performed in the negative mode under the following conditions: mass spectral range, 100–800 *m/z*; event time, 0.2 sec; nebulizing gas (N_2_) flow, 1.5 L/min; drying gas (N_2_) flow, 10 L/min; heat block, 300 °C; CDL temperature, 300 °C. The chia sample was analyzed in triplicate and a semi-quantification was performed by comparing the signal intensity obtained by PDA detection at 280 nm, considering the almost equal UV response to the identified total polyphenols.

### 2.10. Carotenoid Analysis 

Carotenoids were extracted from chia oil by liquid-phase distribution (LPD) between DMF and Hex, according to a slightly modified method reported by Minguez-Mosquera et al. (1992) [43]. Briefly, 1 g of chia oil was dissolved in 6 mL of DMF and treated with five subsequent 2 mL aliquots of Hex, into a decanting funnel. The polar components and the xanthophylls were preserved in the DMF phase, while lipids and carotenes were extracted in the Hex phase. Then, the five Hex extracts were combined, dried by means of a rotary evaporator at 30 °C, reconstituted in 1 mL of Hex, and filtered through a 0.45 µm Acrodisc nylon membrane (Pall Life Science, Ann Arbor, MI, USA) for the HPLC-PDA analysis. The pooled DMF extracts were treated with a 2% Na_2_SO_4_ solution at 0 °C under stirring for 15 min, prior to be transferred in a decanting funnel for extraction with 20 mL mixture of Hex/ethyl ether (1:1, *v/v*). The aqueous phase was discarded, thus eliminating the polar components, whereas the organic phase was evaporated to dryness using the rotary evaporator at 30 °C. The dry residue was dissolved in 1 mL of MeOH/MTBE (1:1, *v/v*) and analyzed by RP-LC-PDA-APCI-MS. The same instrumental setup and software employed for polyphenols (Section 2.9) were used.

Carotenoids chromatographic separations were performed on a YMC C30 column (250 × 4.6 mm, L. × I.D., 3.0 µm *dp*), under gradient elution mode (0–20 min, 0% B; 20–130 min, 0–90% B; 130–140 min, 90–100% B) of (A) MeOH/MTBE/H_2_O (86:12:2, *v/v/v*) and (B) MeOH/MTBE/H_2_O, (8:90:2, *v/v/v*) mobile phases at a flow rate of 0.8 mL/min. The injection volume was 20 µL and the oven was set at room temperature. The UV–Vis spectra were acquired by using deuterium and tungsten lamps in the wavelength range of 250–700 nm and the chromatograms were extracted at 450 nm; the sampling frequency was 1.5625 Hz. MS acquisitions were performed by using an APCI interface in both positive and negative mode under the following conditions: mass range, 350–1200 *m/z*; event time, 1 sec; interface temperature, 350 °C; CDL temperature, 300 °C; heat block temperature, 300 °C; nebulizing gas (N_2_) flow 4 L/min; detector voltage, 0.8 kV. Chia samples were analyzed in triplicate for carotenoid determination. 

Carotenoid quantification was carried out from the calibration curve attained using lutein reference material at six concentration levels in the range between 1 and 200 mg/L (y = 2233x − 42.22; R^2^ = 0.9962).

## 3. Results

Chia (*Salvia hispanica* L.) seeds were extracted in triplicate following the methodology described in Section 2.2, with an average extracted oil yield of 32%.

### 3.1. Total Fatty Acids (FA)

The total FA composition of Chia seed oil analyzed by GC-FID and GC-MS is presented in Table 1 and the GC-FID chromatogram is shown in Figure S1. 

A total of 26 FAs, prior esterification in FAMEs, were identified by using a dual-filter identification strategy, considering the MS similarity (≥850/1000) and comparing experimental (LRI_exp_) and tabulated (LRI_tab_) LRIs for each compound (±10). 

The most abundant FAs is alpha-linolenic acid (C18:3n3) with a percentage of 62.16% (±0.04%), followed by linoleic acid (C18:2n6) with a percentage of 19.26% (±0.00%). Palmitic acid (C16:0) and oleic acid (C18:1n9) showed a similar content in Chia seed oil, accounting for 6.70% (±0.03) and 6.42% (±0.01), respectively, while stearic acid (C18:0) accounted for 3.45% (±0.01).

The total amount of the other FAs is 2.01% and the most representative among these are vaccenic acid (C18:1n7) and arachidic acid (C20:0), with a content of 0.77% (±0.01) and 0.29% (±0.00), respectively.

FA classes, FA ratios, and the nutritional indices, namely AI, TI, and h/H, are reported in Table 2.

From a nutritional point of view, the most interesting results were observed for PUFA content, which represented 81.54% of the total FA composition, and for the quite low percentage of SFAs (11.05%). Then, a favorable PUFA/SFA ratio of 7.42 was calculated, as well as a very low SFA/UFA ratio of 0.12. 

With regard to AI, TI, and h/H indices, strictly related to the FAs profile, AI and TI showed appropriate low values (0.08 and 0.05, respectively), while the h/H ratio of 13.04 was positive as well. 

### 3.2. Free Fatty Acids 

A SPE fractionation was applied to isolate FFAs from Chia seed oil. An amount of 2.1 (±0.1) mg of FFAs were obtained from 60.0 (±0.1) mg of oil. 

**Figure 1:**
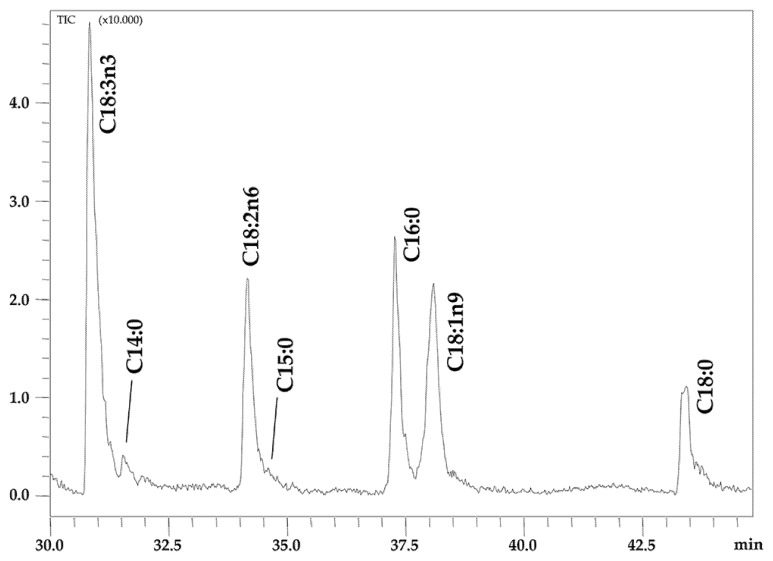
Expansion of NanoLC-EI-MS chromatogram of FFAs in Chia seed oil.

The NanoLC-EI-MS chromatogram of isolated FFAs in Chia seed oil is shown in Figure 1 and the individual amounts of identified FFAs are reported in Table 3.

The identification of seven FFAs was carried out in an automatic manner by matching the acquired EI-MS spectra with those contained in commercial and home-made libraries, the latter built by using the same prototype NanoLC-EI-MS system used for the analysis of the sample, under the same operating conditions. A spectral similarity between 75% and 80% was achieved against commercial libraries, while values up to 97% were obtained when using the home-made database. 

The most representative FFA was C18:3n3 at the percentage of 38.30%, followed by C18:1n9 (22.70%), C18:2n6 (18.19%), C16:0 (13.00%) and C18:0 (6.91%). Traces of C14:0 and C15:0 were quantified at 0.54% and 0.36%, respectively.

UFAs represented almost 80% of the FFA classes with the highest percentage of PUFAs (56.49%), while PUFA/SFA and SFA/UFA ratios were 2.71 and 0.26, respectively. n3-PUFA content is wholly represented by C18:3n3 (38.30%), while n6-PUFA content is only represented by C18:2n6 (18.19%). 

AI and TI indices showed similarly low values (0.19 and 0.13, respectively) and the h/H ratio was 5.85.

### 3.3. Triacylglycerols

**Figure 2:**
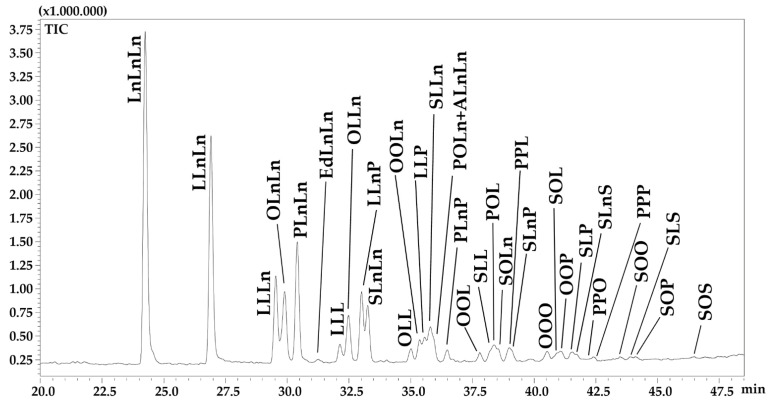
Expansion of NARP-HPLC-APCI(^+^)-qMS chromatogram of TAGs in Chia seed oil.

The NARP-HPLC-APCI^(+)^-qMS chromatogram of TAGs in Chia seed oil is shown in Figure 2. Actually, NARP-HPLC methods enable the detection of the entire acylglycerol fraction, including mono- and diacylglycerols in the first region of the chromatogram. However, no peaks were detected prior than 20 min of elution time. The identified and quantified TAGs, for a total of 34 non-polar lipids, are listed in Table 4 according to the conventional notation that refers to the abbreviation of FAs’ names ordered according to their decreasing molecular weights [36,37]. Table 4 also shows the total carbon number (CN) of all acyl chains, the number of double bonds (DBs), the partition number (PN) defined as PN = CN−2DB, and the average % area, corrected by response factors, that allowed a relative semi-quantification of identified TAGs (three replicates). 

Identified TAGs contain 7 different FAs, namely palmitic acid (C16:0), heptadecanoic acid (C17:0), stearic acid (C18:0), oleic acid (C18:1n9), linoleic acid (C18:2n6), α-linolenic acid (C18:3n3) and arachidic acid (C20:0). The most abundant components were: trilinolenin (LnLnLn) at 17.73% (±0.43); dilinoleoyl-linoleoyl glycerol (LLnLn) at 13.35% (±0.34) and dilinoleoyl-palmitoyl glycerol (PLnLn) at 10.56% (±0.48), accounting for more than 40% of the total composition.

The remaining percentage, just under 60%, corresponds to TAGs with individual percentages ranging from roughly 2% to 7% (LLLn, OLnLn, OLLn, LLnP, SLnLn, OOLn, LLP and SLLn) and minor TAGs with individual percentages under 2%.

### 3.4. Phospholipid

PLs were isolated by the SPE procedure (1.0 (±0.1) mg of PLs were obtained from 60.0 (±0.1) mg of oil and analyzed by RP-UHPLC-APCI^(+/−)^-qMS as described in Section 2.6.

**Figure 3:**
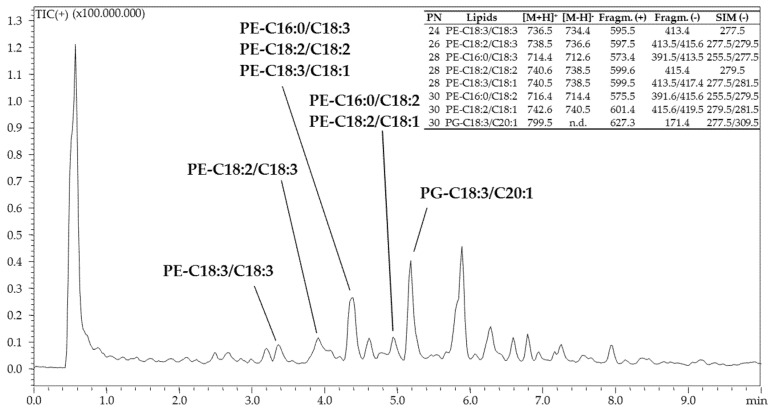
RP-UHPLC-APCI^(+)^-qMS analysis of phospholipids in Chia seed oil.

The chromatogram obtained for the analysis of PLs is shown in Figure 3. 

Identified PLs are reported in the table inserted inside Figure 3. A total of 8 PLs were identified in PNs ranging from 24 to 30, consisting of 7 phosphoethanolamines (PEs) and one phosphatidylglycerol (PG). Identification of the PE class was confirmed by protonated [M + H]^+^ and deprotonated [M − H]^−^ molecular ions supported by characteristic PE fragment ions under APCI-MS in positive [M + H − C_2_H_8_NO_4_P]^+^ = [M + H − 141]^+^ (loss of the polar headgroup) and negative [M-H-R_sn_-_1/2_COOH]^−^= [M-H-FA_sn_-_1/2_]^−^ (loss of FA) modes.

The only PG detected (PG-C18:3/C20:1) was identified by combining the protonated [M + H]^+^ (*m/z* 799.6) and the PG characteristic fragment ions under APCI-MS in positive [M − C_3_H_7_O_2_OPO_3_H]^+^ = [M − 171]^+^ (loss of the polar headgroup) and in negative [C_3_H_7_O_2_OPO_3_H]^−^ (*m/z* 171.4, glycerol phosphate anion).

As additional filter identification, several ions related to the loss of FAs from the PLs structure were monitored (Figure 3). A more detailed explanation about analytical methodology, PLs fragmentation, and identification criteria is out of the scope of the present work and further information can be found in the corresponding literature [38].

### 3.5. Sterols

Sterols determination was carried out through the official method UE N. 1348/2013 [39], as described in Section 2.7. Identification was performed by comparing their relative retention time, calculated against the retention time of dehydrocholesterol as IS, with the elution profile reported in the official procedure [39]. A total of 8 sterols were identified and quantified. The predominant compound was β-sitosterol at a percentage of 68.54% (±1.80). Campesterol, Δ-5-avenasterol, and stigmasterol were found at 12.23% (±0.54), 8.93% (±1.32), and 4.30% (±0.20), respectively. A smaller percentage was represented by Δ-5-2,4 stigmastanol, Δ-7-avenasterol, Δ-7-stigmastanol, and clerosterol. The former accounting for around 2.50%, while the others account for around 1.50% each.

### 3.6. Bioactive Compounds (Tocopherol, Polyphenol, Carotenoid)

A total tocopherol (vitamin E) amount of 22.88 μg/mL was quantified by three replicates of HPLC analysis with fluorimetric detection, using a validated method for each tocopherol as described in Section 2.8. An example of the obtained chromatogram of tocopherols in Chia seed oil is shown in Figure S2. 

Among the vitamin E components, γ-tocopherol was the major compound at the amount of 15.30 μg/mL (±0.61), followed by δ-tocopherol at 5.79 μg/mL (±0.08) and α-tocopherol at 1.79 μg/mL (±0.17).

**Figure 4:**
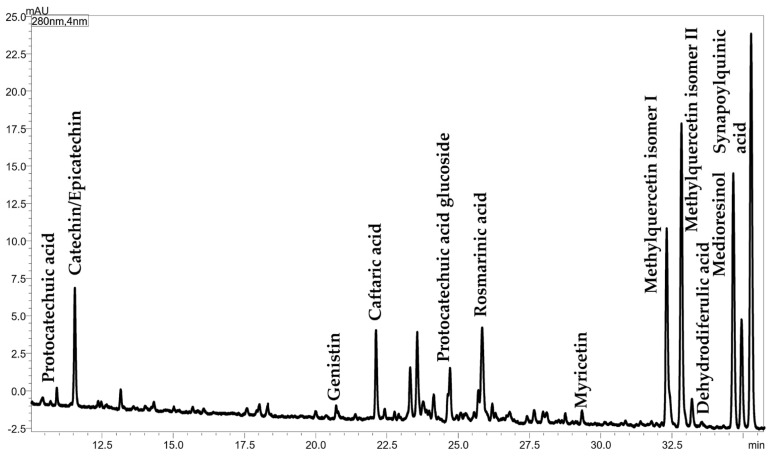
Expansion of the RP-LC-ESI(^−^)-qMS chromatogram of polyphenols in Chia seed oil.

Phenolic compounds were identified by RP-LC-ESI(^−^)-qMS analysis and the obtained chromatogram, along with peak identification, is reported in Figure 4.

A total of 12 compounds, reported in Table S1, were identified by using the molecule-related ion information [M-H]^−^ and confirmed by using two databases (http://phenol-explorer.eu/downloads, accessed on 7 December 2022; https://hmdb.ca/spectra/ms/search, accessed on 7 December 2022) in a mass range from 153.1 to 431.2 *m/z*. Six compounds belong to the phenolic acid chemical class, mainly to the hydroxycinnamic acid family, apart from protocatechuic acid (*m/z* = 153.1) which is a benzoic acid derivative. Three flavonols, namely myricetin (*m/z* = 317.5) and two isomers of methylquercetin (*m/z* = 315.3), were identified. A catechin (*m/z* = 289.3), belonging to the flavonol class, was also detected, as well as the isoflavone glucoside genistin (*m/z* = 431.2) and the lignan medioresinol (*m/z* = 387.3). Among them, one isomer of methylquercetin was the most intense peak of the chromatogram.

Carotenoids extraction from chia oil provided two fractions of different polarity, resulting from a liquid phase distribution in two solvents. Thus, polar carotenoids, namely xanthophylls were contained in the polar DMF phase, while non-polar carotenes were dissolved into the n-hexane phase. The only trace of β-carotene was detected in the non-polar phase.

As for the polar phase, a total of 8 xanthophylls were identified, based on complementary information consisting of their retention behaviors under RP conditions (elution order in correlation with the chemical structure), UV–vis, and MS spectral data. Figure S3 reports the chromatographic separation of xanthophylls in chia seed oil and Table S2 reports peak identification along with spectral data. The isomeric structures of zeaxanthin and lutein (all-*E* isomers) appeared as the most intense signals.

## 4. Discussion

In the last years, there has been an increased interest in chia seeds in both human and animal nutrition. *Salvia hispanica* L. seeds, in Europe, are classified as a novel food [29], and they are considered as new foodstuffs with health-promoting qualities because they are a good source of dietary fiber and present a high content of ω-3.

Chia seeds are usually used in the form of flour, oil, or whole seeds. The content and composition of the oil extracted from chia seeds depend on the origin of the plant, the climatic conditions of the growing location, and the extraction technique. The oil yield usually ranged between 29.4% and 33.5% [21,44], for other chia seed samples coming from Ecuador and Chile South-America regions, showing a negligible variability by changing the cultivation area. Given that the chia seed oil yield obtained in this trial (32%) was in agreement with such range, a comprehensive characterization of the lipid fraction of this sample can be considered representative. Such a characterization of the lipid fraction of chia seed oil has never been done before. In fact, most of the reported work in the literature focused on a few major or minor constituents such as FAs, TAGs, PLs, sterols, polyphenols, carotenes, and tocopherols [14,27,45,46,47], while FFAs and xanthophylls have never been investigated.

Among the high levels of lipids, the chia seed oil analyzed in this case resulted rich in ω-3, ω-6, and ω-9 FAs. These accounted for 62.20%, 19.27%, and 6.47% of the total lipids, respectively. In agreement with data previously reported, chia seed oil is expected to contain more than 60% of ω-3 alpha-linolenic acid (ALA) and 15% of ω-6 linoleic acid [12,27]. These results confirm that chia seeds contain the highest concentration of ω-3 and ω-6 FAs among all known food sources [3,22].

The beneficial health effect of the consumption of functional foods containing high amounts of PUFAs is well recognized [12]. Moreover, the American Heart Association recommends the use of unsaturated oils instead of saturated fats to prevent cardiovascular diseases, hypertension, obesity, diabetes, and other health-related disorders [48]. Therefore, because of the high concentration of PUFAs in chia seed oil, its demand has severely increased.

Results obtained for saturated FAs in chia seed oil obtained in this work (Table 1), were in accordance with previous literature findings, reporting the total SFA content at a percentage in the range 9–12%, with significant variation depending mainly on the geographical origin [12,27,49]. On the other hand, a lower content of oleic acid was obtained in the present study compared to the work by Coelho and Salas-Mellado (2014) [27]. However, this was counter-balanced by an increase of the content of PUFAs. 

The ratios between FA classes (SFA, PUFA, MUFA) provide immediate information about the contribution of such edible oil in the context of a balanced diet. This is certainly more interesting from a nutritional point of view than discussing the total amount of the single FA classes. In this regard, an SFA/MUFA ratio higher than the most traditional vegetable oils (olive, soybean, sunflower, corn, etc.) was determined. This is due to the lower content of oleic acid. However, this is counterbalanced by a considerably higher PUFA/SFA ratio (7.37, Table 2). This is not only true with respect to the above-mentioned vegetable oils but also when compared to some less common seed oils, such as hempseed (~6.5 according to Arena et al. (2022) [50]) and canola (~3.4, as reported by Atefi et al. (2018) [51]) oils. Moreover, the PUFA/SFA ratio found is also higher than fish and algae oils, which are characterized by a significantly higher SFA content, despite being remarkably interesting due to their high content of n3 PUFAs and poor level of n6 PUFAs [52]. Moreover, an extremely favorable SFA/UFA ratio, equal to 0.12, was obtained for the analyzed chia oil. This ratio is even comparable to the most common vegetable oils, which present SFA/UFA ≥ 0.16 for olive, corn, and soybean oils, while a range of 0.11–0.16 can be found for sunflower oil [51,53].

Within the PUFA fraction, the distribution between n3 and n6 FAs can be directly correlated to some beneficial properties of the oil. In particular, a favorable n3/n6 ratio (>1) is extremely beneficial for human health, since ω-3 PUFAs are precursors of anti-inflammatory molecules, which are active in the reduction of platelet aggregation, coagulation, and thrombosis [54,55]. An n3/n6 ratio ≤ 0.1 is found in the most common seed oils due to the predominance of linoleic acid and almost absence of ALA (e.g., sunflower << corn < soybean oil [53]. Moreover, n3/n6 ratio is lower than 1 in most uncommon vegetable oils, such as canola oil (~0.2 according to Subash-Babu and Alshatwi (2018) [54]) and hempseed oil (~0.25 according to Arena et al. (2022) [50]) despite they are recognized as an ω-3 source. The chia seed oil characterized in this study showed a 3.23 n3/n6 ratio, comparable to flaxseed oil, but lower than some fish and algae oils, as previously suggested [52]. 

In order to consider the combination between all these ratios, three indices were calculated and reported in Table 2: atherogenic index (AI), thrombogenic index (TI), and the ratio of hypocholesterolemic and hypercholesterolemic FAs (h/H). AI represents the ratio between the sum of some SFAs and UFAs, removing stearic acid (C18:0) from SFA since it is not considered an atherogenic acid according to experimental evidence [56]. To prevent the risk of atherosclerosis, AI values close to zero are preferable. In this study, AI was 0.08, a value smaller than the one reported for olive oil (AI = 0.14), marine organisms, and seaweeds (high variability depending on the species), and similar to the value reported for sunflower, and flaxseed oil (AI = 0.07, for both oils) [33,56,57], and more recently for hempseed oil (AI = 0.07–0.08) [50].

Concerning TI, values close to zero indicate a low risk of thrombus formation. Its calculation considers different multiplying factors for the UFA classes, among which ω-3 species provide the highest contribution in the prevention of thrombosis. In this study, TI showed a value of 0.05, which is far smaller than the values obtained for olive, sunflower, and hempseed oil (TI = 0.32, 0.28, 0.11–0.14, respectively) [50,56], as well as marine organisms (TI = 0.16 for mackerel and up to 0.74 for tuna) [33], and a slightly lower than linseed oil (TI = 0.07) [57] due to the highest percentage of ALA.

Finally, the h/H ratio was calculated to estimate the hypocholesterolemic effect of chia oil. It resulted in 13.04 and it was significantly higher than the ratio obtained for marine organisms [33] and common vegetable oils [53], due to the lower content of palmitic acid. Nevertheless, it fell into the range of 11.5–14.0 reported for hempseed products [50], and it was smaller than the value obtained for flaxseed oil (h/H = 14.75) [57]. 

Similar considerations were applied in the study of FFAs, which represent a minor lipid fraction readily available to take part in metabolic processes. However, from an analytical point of view, in contrast to the esterified FA composition that was elucidated through conventional GC-FID/MS analyses, FFAs were determined through a prototype NanoLC-EI-MS system which uncommonly combines an LC platform with EI-MS detection, normally coupled to GC. Such a prototypeenables their analyses without the need for derivatization procedures, normally required prior to a GC analysis due to the polar nature of the free carboxylic group. The peculiar coupling between a miniaturized LC system and an MS detector equipped with an EI source allowed for a fast, automatic, and reliable identification of FFAs in chia seed oil. In fact, the use of the commercial or home-made spectral database was helpful to achieve a univocal peak assignment. However, despite the well-known high reproducibility of EI-MS spectra, the spectral similarity achieved against commercial libraries was lower than usual. This is probably due to a more expressed molecular ion in the LC-EI spectra because of the use of a protic solvent (mixture H_2_O/ACN) that stabilized the molecular cationic radical. Therefore, the building of a home-made database greatly increased the spectral similarity. 

In comparison with the esterified FA composition (Table 1), only major FAs were detected as not esterified species (Table 3), being ALA the major component. The smaller percentage of ALA in this case compared to the one obtained when analyzing the esterified FA composition (38.30% against 62.16%), indicates that most ALA is esterified to glycerol. Conversely, oleic acid presented a significantly higher percentage of the FFA fraction than of the esterified FA portion (22.70% against 6.42%), meaning that a high proportion of such FA is present as FFA. Similarly, palmitic and stearic acids were detected at levels twice higher as FFA than the percentages calculated with respect to the esterified FA composition. Interestingly some FFAs that were not detected in either TAGs or PLs were found during this analysis, namely C14:0 and C15:0 (viz. esterified FA composition previously discussed). This is likely due to the fact at these medium-long chain FAs, although present in small amounts, exist only as free FAs. Thus, they never esterified to glycerol and therefore will never form either storage fats or membrane lipids, but they could be used for elongation processes in order to form other FAs.

All these differences between FFAs and esterified FAs are reflected in the calculated nutritional indices. For instance, a lower PUFA/SFA ratio was obtained for FFA, which can negatively affect the quality of chia seed oil. However, the SFA/MUFA ratio resulted smaller, which is extremely positive from a nutritional point of view. Nevertheless, no correlation can be made with other vegetable oils since the FFA fraction is normally neglected by researchers, probably due to difficulty in its isolation and analysis. Hopefully, this can open new insights in the evaluation of the nutritional quality of an oil.

Among intact lipids, the TAG analysis was carried out by NARP-HPLC-APCI(^+^)-MS obtaining a satisfactory separation and detection of single molecular species. Triacylglycerols are eluted according to increasing PN. Within PN group, the FA position into the glycerol backbone affects the TAG elution order, even in the case of regioisomeric species. For instance, the elution order of *m/z* 880 isomeric TAGs at PN42, where the retention of LLL (C_18:2_C_18:2_C_18:2_) < OLLn (C_18:1_C_18:2_C_18:3_) < SLnLn (C_18:0_C_18:3_C_18:3_) (Figure 2). Such a regular chromatographic profile is helpful in the identification process, especially in the case of minor peaks often presenting a noisy MS spectrum, so that the retention data can drive the correct peak assignment.

Half of the identified TAGs contained at least a molecule of ALA (Table 4), according to the results previously discussed for the esterified FA and FFA compositions. Most of them eluted at low PN regions and correspond to the most intense signals. Little information about the characterization of TAGs in chia seed oil exists in the literature; only 12 TAGs identified and quantified in this study have been determined in a previously published work [12], dealing with chia seed oil coming from two different South America locations (Argentina and Guatemala) extracted with two different extraction processes (solvent vs pressing). In particular, the extraction method did not affect the TAG composition, while significant differences were encountered depending on the growing conditions.

Aside from TAGs, which are storage fats, the minor PL fraction (around 1.6% of the chemical composition of the oil analyzed immediately after the Soxhlet extraction), which is involved in different biological processes, was also investigated. Although the chemical composition of chia seed oil of European origin has been investigated in a previous work [58], to date, there are no information about the PLs fraction of chia seeds coming from other countries. This is mostly because the PL content is concentrated in the residue (not considered in this work) rather than in the oil obtained after soxhlet extraction with n-hexane. In fact, the oil analyzed in the previous work [58] was extracted by Folch method, which provides a more comprehensive lipid fraction, including both polar and apolar components. Similarly to TAGs, PLs eluted according to their PN under RPLC mode. However, the retention is affected not only from the FA position in the glycerol backbone, but also from the polar head-group, characteristic for each PL class. Thus, under RPLC, PLs belonging to different classes but with the same PN may have different retentions [38]. Apart from a PG (PG_C18:3/C20:1_), the only PL class detected was the PE. As for the other lipid classes, the most abundant FA in the PL fraction was ALA, present in 5 out of 8 PLs identified (Figure 3).

The high content of PUFAs makes the oil highly susceptible to oxidation reactions. For this reason, the analysis of antioxidant molecules, such as phenolic compounds and tocopherols, is mandatory to evaluate the stability of the oil. Tocopherols are lipid-soluble compounds also known as vitamin E. They are capable to protect oils from oxidation, and high amounts of tocopherols are usually related to a high PUFA content [12]. In general, α-tocopherol is the most representative vitamer in olive oil, while seed oils contain higher levels of γ- and δ-tocopherols, which have been reported to exert a synergistic antioxidant action with α-tocopherol, which is employed in many dietary supplementations [59]. In fact, the study of the antioxidant mechanism highlighted that α-tocopherol alone, despite having a faster radical scavenging activity, has a certain prooxidant effect due to the formation of a less stable phenolic radical. Then, the coexistence in the food product of other vitamers enhance the antioxidant effect, thus efficiently preserving lipids from peroxidation processes. Within this context, the chia seed oil analyzed in this study through a conventional HPLC analysis, showed a greater amount of γ- and δ-tocopherols compared to the α vitamer. This was also in partial accordance with previous findings highlighting γ-tocopherols as the main form of vitamin E, accounting for more than 85% of the vitamin E fraction, while β-tocopherol has never been detected [12,46]. In our study, a higher percentage of δ-tocopherol was quantified, so that the sum of γ and δ-tocopherols represented 92% of the total vitamin E amount. To this regard, differences in the vitamin E composition can be related to both geographical origin and extraction methods, as highlighted by Ixtaina et al. [12] for Argentina and Guatemala seeds extracted by solvent and pressing techniques, and Dąbrowski et al. [46] for 15 chia seed oil samples originating from five South American countries and extracted by Soxhlet.

Phenolic compounds are other minor bioactive components in chia seeds and oil, responsible for their antioxidant potential, which reduce the risk of cardiovascular disease and have hepatoprotective effects, as well as act against oxidative stress and obesity-related diseases [60]. It is well known that the production of phenolic compounds depends on biotic and abiotic stress conditions to which the plant is exposed [61]. Therefore, different phytochemical compositions can be obtained by changing the cultivation area and pedoclimatic conditions, as also reported for chia seed oils [12]. In this work, 12 phenolic compounds were identified by considering the retention data in combination with the mass spectrum, which provided the mass of the deprotonated molecule. Online databases were used to create a list of possible candidates for each detected ion, while previously published papers were used to select the most probable compound. Only 5 out of 12 compounds have been already reported in the literature, namely the phenolic acid protocatechuic acid and rosmarinic acid, the flavanol epicatechin, the isoflavone glucoside genistin and the flavonol myricetin [12,60,62,63]. Other compounds were selected since they are structurally related to previously identified molecules, such as protocatechuic acid glucoside, two isomers of methylquercetin, and derivatives of hydroxycinnamic acid. The identification of two quercetin derivatives is in accordance with the findings of Ixtaina et al. (2011) [12] and Marineli et al. (2015) [60], who detected quercetin as one of the three identified flavonols (myricetin, quercetin, and kaempferol, the latter not detected in this study). As for hydroxycinnamic acid derivatives, Oliveira-Alves et al. (2017) [62] listed them as the main phenolic compounds in chia seed products. In this study, four hydroxycinnamic acid derivatives were identified as monomers (caftaric acid and sinapoylquinic acid) and dimers (rosmarinic acid and dehydrodiferulic acid). 

Finally, among antioxidants, a class of natural pigments were investigated in this work, namely carotenes and xanthophylls. They belong to the carotenoid family, being the first non-polar compounds and the latter polar molecules. To the best of our knowledge, previously reported work focused on the quantification of β-carotene, with the exception of a single scientific work [46] in which some polar carotenoids were detected but not identified, meaning that they were reported as unidentified carotenoid-like compounds. Then, this is the first work reporting the identification of xanthophylls in chia seed oil. They were identified by exploiting the complementarity between the MS spectrum, UV-Vis data, and their retention behavior under RP conditions. Particularly, MS data including both the molecule-related-ion and the fragments characteristics of the functional group bound to the molecules (i.e., epoxide, hydroxyl, methyl, ethyl, etc.), were helpful for structure elucidation purposes. In addition, *cis* and *trans* isomers were distinguished through the UV-Vis absorption bands. In fact, the isomer of Zeaxanthin with a *cis*-bond, instead of all-*trans*, shows an additional absorption band at λ = 338 nm.

Lastly, the phytosterol fraction was investigated in the present work as a particularly interesting minor lipid class, which can be usefully correlated with seed origin [46], method of extraction [64] and quality of the refining process, which normally determine a loss of sterols [65]. In this study, eight phytosterols were identified, with β-sitosterol as the most abundant sterol, accounting for more than 68% of the total amount of sterols. It was followed by campesterol (12.23%) and Δ-5-avenasterol (8.93%). Few scientific research studies on sterol content in chia oil are present in the literature, and only 3 or 4 compounds have been identified [4,28,48,64,65,66,67], with a variable range of relative concentrations. Therefore, a more in-depth study of the identification and quantification of sterols in chia oils obtained from different varieties of chia is still needed to establish the concentration range according to different parameters such as the cultivation area or the extraction method of the oil.

## 5. Conclusions

A combination of LC techniques was used to determine the comprehensive oil composition, including both main components such as triacylglycerol, and bioactive components such as tocopherols, polyphenols, and carotenoids. These LC techniques involved the combination of different sample pre-treatments (solvent extraction, SPE) and detectors (PDA, MS, fluorescence). 

Moreover, the use of SPE fractionation of the oil in polar, mid-polar, and non-polar fractions, followed by their characterizations using advanced analytical methodologies such as nanoLC-EI-MS, could be the first step to build an online 2D-LC platform for simultaneous characterization of all the fractions in chia oil

Thanks to the combination of this myriad of analytical techniques, the chia seed oil was characterized in detail for the first time. Results exceeded the currently available information as in the case of FFAs and polar carotenoids, which were reported for the first time. The studied sample presented a high content of essential FAs and a conspicuous number of bioactive compounds. In addition, ratios of different compounds were used to assess the nutritional value and they were compared to other largely studied samples. Very beneficial SFA/UFA and n3/n6 ratios were found. Moreover, atherogenic and thrombogenic indexes were extremely favorable. However, the hypocholesterolemic and hypercholesterolemic index was higher than that of common vegetable oils and marine organisms. Therefore, this comprehensive investigation did not only allow a characterization in terms of major and minor constituents, but also in terms of the nutritional value of chia seed oil. 

## Figures and Tables

**Table 1:** Mean content of FAs in Chia seed oil, determined as FAMEs by GC-FID/MS, along with MS similarity and LRI values, compared to tabulated LRI.

Compound Name	Symbol	% ± SD (n = 3)	LRI_exp_	LRI_tab_	Similarity
Myristic acid	C14:0	0.04 ± 0.00	1401	1400	96%
Pentadecanoic acid (anteiso)	C15:0 anteiso	0.01 ± 0.00	1468	1468	90%
Pentadecanoic acid	C15:0	0.02 ± 0.00	1498	1500	95%
Palmitic acid	C16:0	6.70 ± 0.03	1601	1600	97%
Hexadecenoic acid	C16:1n9	0.02 ± 0.00	1620	1622	94%
Palmitoleic acid	C16:1n7	0.06 ± 0.00	1623	1624	98%
Heptadecanoic acid (anteiso)	C17:0 anteiso	0.17 ± 0.00	1668	1668	96%
Hexadecadienoic acid	C16:2n4	0.01 ± 0.00	1681	1687	90%
Heptadecanoic acid	C17:0	0.04 ± 0.00	1699	1700	96%
Heptadecenoic acid	C17:1n7	0.02 ± 0.00	1724	1728	93%
Octadecanoic acid (iso)	C18:0 iso	0.04 ± 0.00	1745	1750	95%
Stearic acid	C18:0	3.45 ± 0.01	1800	1800	97%
Oleic acid	C18:1n9	6.42 ± 0.01	1822	1819	97%
Vaccenic acid	C18:1n7	0.77 ± 0.01	1829	1824	98%
Linoleic acid	C18:2n6	19.23 ± 0.00	1867	1864	97%
Nonadecanoic acid (anteiso)	C19:0 anteiso	0.06 ± 0.00	1870	1867	96%
α-linolenic acid	C18:3n3	62.16 ± 0.04	1925	1928	96%
Eicosanoic acid (iso)	C20:0 iso	0.01 ± 0.00	1953	1953	88%
Arachidic acid	C20:0	0.29 ± 0.00	2002	2000	96%
Eicosenoic acid	C20:1n7	0.13 ± 0.00	2020	2016	96%
Eicosadienoic acid	C20:2n6	0.04 ± 0.00	2061	2068	94%
Heneicosanoic acid	C21:0 iso	0.05 ± 0.00	2066	2058	91%
Eicosatrienoic acid	C20:4n3	0.04 ± 0.00	2117	2118	88%
Docosanoic acid	C22:0	0.08 ± 0.00	2195	2200	95%
Tricosanoic acid	C23:0	0.03 ± 0.00	2295	2300	94%
Lignoceric acid	C24:0	0.11 ± 0.01	2398	2400	92%

SD: Standard deviation.

**Table 2:** Mean values of three replicates of fatty acid classes, ratios, and nutritional indices in Chia seed oil.

Symbol	Average Value
SFA	11.05%
MUFA	7.42%
PUFA	81.48%
SFA/MUFA	1.49
PUFA/SFA	7.37
SFA/UFA	0.12
n3	62.20%
n6	19.27%
n3/n6	3.23
AI	0.08
TI	0.05
h/H	13.04

SFA = saturated fatty acids; MUFA = monounsaturated fatty acids; PUFA = polyunsaturated fatty acids; n3 = n3-polyunsaturated fatty acids; n6 = n6-polyunsaturated fatty acids; SFA/MUFA = saturated/monounsaturated fatty acid ratio; PUFA/SFA = polyunsaturated/saturated fatty acid ratio; SFA/UFA = saturated/unsaturated fatty acid ratio; AI = atherogenic index; TI = thrombogenic index; h/H = hypo-/Hypercholesterolemic ratio.

**Table 3:** Mean content of identified FFAs, along with their MS similarity, FFA classes and ratio, and nutritional indices in Chia seed oil.

FFA	Compound	MS Similarity	% ± SD (n = 3)
C18:3n3	α-linolenic acid	79% ^1^, 93% ^2^	38.30 ± 3.04
C14:0	Myristic acid	80% ^2^	0.54 ± 0.13
C18:2n6	Linoleic acid	80% ^1^, 92% ^2^	18.19 ± 1.57
C15:0	Pentadecanoic acid	78% ^2^	0.36 ± 0.08
C16:0	Palmitic acid	81% ^1^, 97% ^2^	13.00 ± 1.58
C18:1n9	Oleic acid	76% ^2^	22.70 ± 1.85
C18:0	Stearic acid	76% ^1^, 91% ^2^	6.91 ± 0.67
	SFA		20.81
	MUFA		22.70
	PUFA		56.49
	SFA/MUFA		0.92
	PUFA/SFA		2.71
	SFA/UFA		0.26
	n3		38.30
	n6		18.19
	n3/n6		2.10
	AI		0.19
	TI		0.13
	h/H		5.85

^1^: Commercial library; ^2^: Home-made library; SFA = saturated fatty acids; MUFA = monounsaturated fatty acids; PUFA = polyunsaturated fatty acids; n3 = n3-polyunsaturated fatty acids; n6 = n6-polyunsaturated fatty acids; SFA/MUFA = saturated/monounsaturated fatty acid ratio; PUFA/SFA = polyunsaturated/saturated fatty acid ratio; SFA/UFA = saturated/unsaturated fatty acid ratio; AI = atherogenic index; TI = thrombogenic index; h/H = hypo-/Hypercholesterolemic ratio.

**Table 4:** Identified TAGs, along with CN, DB, PN and their mean % content in Chia seed oil.

TAGs	CN	DB	PN	% ± SD (n = 3)
LnLnLn	54	9	36	17.73 ± 0.43
LLnLn	54	8	38	13.35 ± 0.34
LLLn	54	7	40	5.50 ± 0.53
OLnLn	54	7	40	6.16 ± 0.26
PLnLn	52	6	40	10.56 ± 0.48
EdLnLn	53	6	41	0.37 ± 0.03
LLL	54	6	42	1.31 ± 0.04
OLLn	54	6	42	4.69 ± 0.43
LLnP	54	5	42	6.98 ± 0.22
SLnLn	54	6	42	3.43 ± 0.21
OLL	54	5	44	1.48 ± 0.23
OOLn	54	5	44	2.43 ± 0.28
LLP	52	4	44	2.42 ± 0.19
SLLn	54	5	44	2.30 ± 0.17
POLn	52	4	44	1.74 ± 0.25
ALnLn	56	6	44	1.70 ± 0.25
PLnP	50	3	44	1.66 ± 0.13
OOL	54	4	46	1.05 ± 0.02
SLL	54	4	46	0.66 ± 0.13
POL	52	3	46	1.97 ± 0.38
SOLn	54	4	46	0.85 ± 0.02
PPL	50	2	46	1.78 ± 0.03
SLnP	52	3	46	0.75 ± 0.04
OOO	54	3	48	1.81 ± 0.27
SOL	54	3	48	0.84 ± 0.08
OOP	52	2	48	1.88 ± 0.19
SLP	52	2	48	0.93 ± 0.04
SLnS	54	3	48	0.29 ± 0.02
PPO	50	1	48	0.44 ±0.06
PPP	48	0	48	1.04 ± 0.08
SOO	54	2	50	0.76 ± 0.07
SLS	54	2	50	0.36 ± 0.02
SOP	52	1	50	0.55 ± 0.05
SOS	54	1	52	0.25 ± 0.04

CN: Carbon number; DB: Number of double bonds; PN: Partition number; SD: Standard deviation; P: Palmitic acid; S: Stearic acid; O: Oleic acid; L: Linoleic acid; Ln: α-linolenic acid; A: Arachidic acid; Ed: Heptadecanoic acid.

## Data Availability

Data is contained within the article and supplementary material.

## Supplementary Materials

The following supporting information can be downloaded at: https://www.mdpi.com/article/10.3390/foods12010023/s1, Figure S1: GC-FID chromatogram relative to the esterified FA composition of chia seed oil; Figure S2: HPLC chromatogram of tocopherols in chia seed oil. The table in the insert report the quantitative results (mg/mL) along with the standard deviation (SD) of three replicates; Figure S3: HPLC-PDA chromatogram, extracted at 450 nm, of xanthophylls in chia seed oil. For peak number, see Table S2. Table S1: List of identified phenolic compounds in Chia seed oil, along with retention time (RT), molecule-related ion ([M-H]-) and chemical class; Table S2: List of identified xanthophylls along with UV-VIS and MS spectral data (molecule-related ion and fragments in positive ion mode).
