# Supplementary material for: Comprehensive Chemical Characterization of Chia (Salvia hispanica L.) Seed Oil with a Focus on Minor Lipid Components
Source: Foods. 2022 Dec 21;12(1):23. doi: 10.3390/foods12010023 (PMC9818636; doi:10.3390/foods12010023)
Supplement: Supplementary file 1 [file foods-12-00023-s001.zip › foods-2051837-supplementary.pdf]

Supplementary Materials

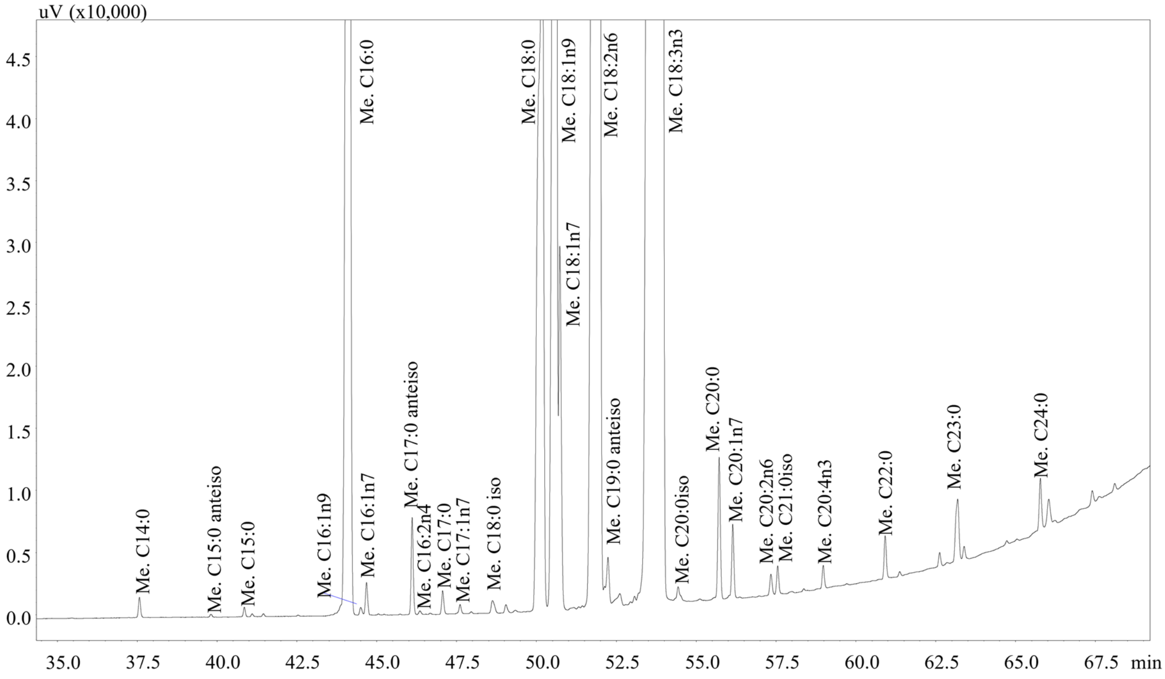

Figure S1: GC-FID chromatogram relative to the esterified FA composition of chia seed oil.

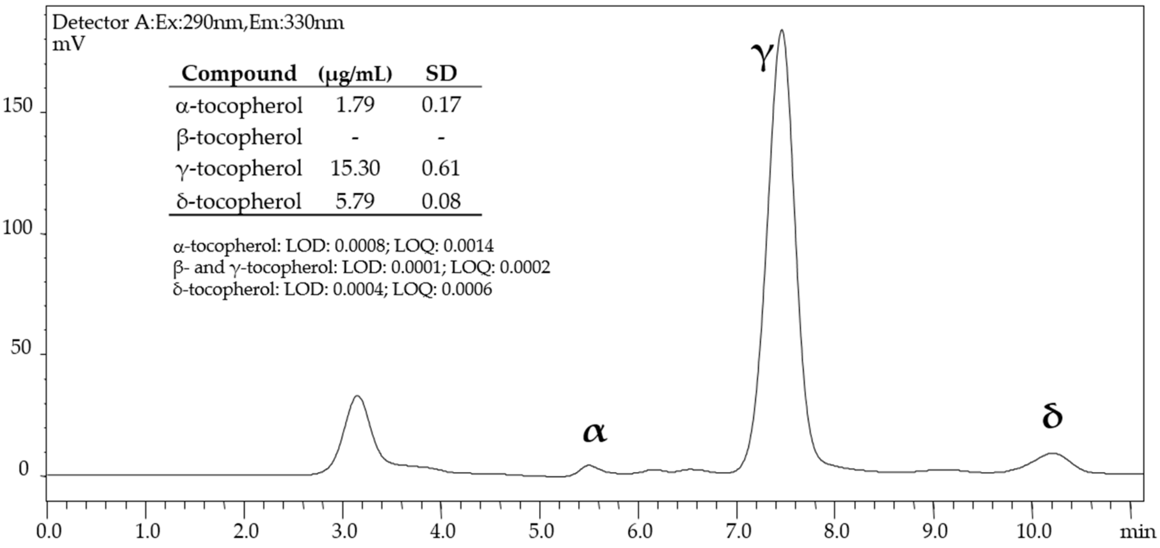

Figure S2: HPLC chromatogram of tocopherols in chia seed oil. The table in the insert report the quantitative results (mg/mL) along with the standard deviation (SD) of three replicates.

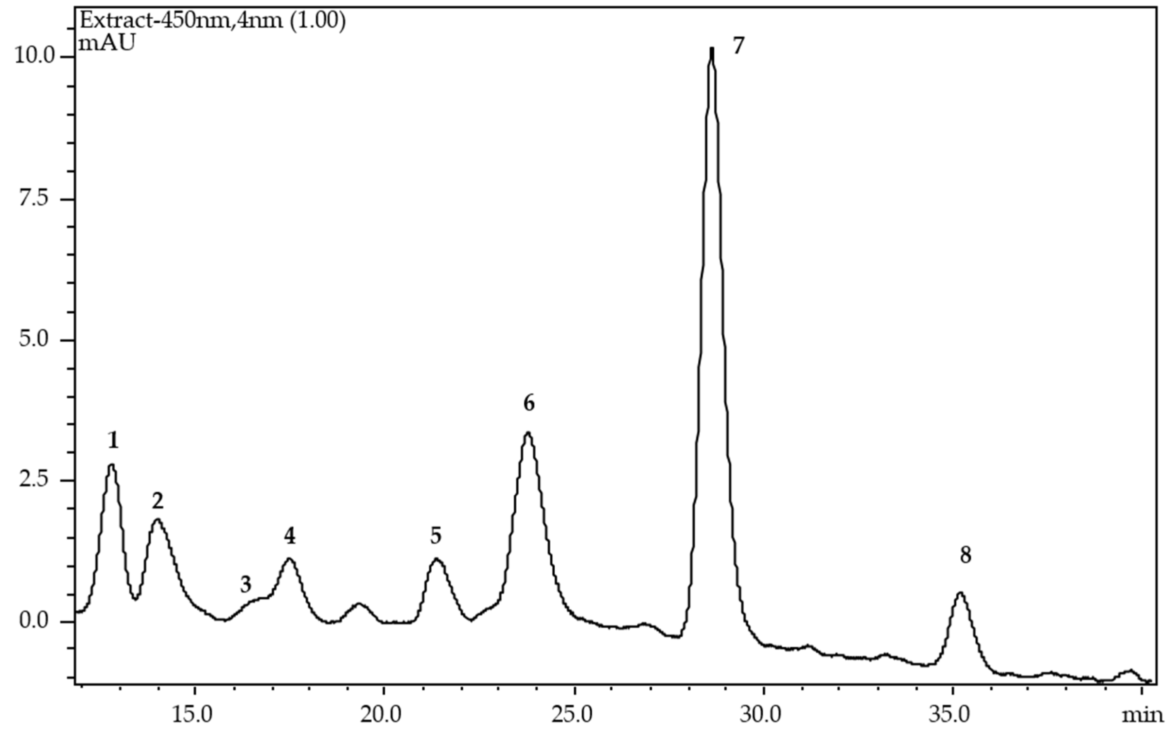

Figure S3: HPLC-PDA chromatogram, extracted at 450 nm, of xanthophylls in chia seed oil. Peak numbering: 1, Neochrome; 2, Luteoxanthin; 3, Auroxanthin; 4, Antheraxanthin; 5, Mutatoxanthin; 6, Lutein; 7, all E-Zeaxanthin; 8, 9,9'Z-Zeaxanthin.

Table S1. List of identified phenolic compounds in Chia seed oil, along with retention time (RT), molecule-related ion ($[M-H]^-$) and chemical class.

RT	$[M-H]^-$	Compounds	Chemical Classes
10.93	153.1	Protocatechuic acid	Phenolic acid (benzoic acid derivative)
11.55	289.3	Catechin/Epicatechin	Flavanol
20.72	431.2	Genistin	Isoflavone glucoside
22.13	311.3	Caftaric acid	Phenolic acid (hydroxycinnamic acid derivative)
24.71	315.3	Protocatechuic acid glucoside	Phenolic acid derivative (phenolic glucoside)
25.85	359.2	Rosmarinic acid	Phenolic acid (hydroxycinnamic acid derivative)
29.35	317.5	Myricetin	Flavonol
32.31	315.3	Methylquercetin isomer I	Flavonol
32.83	315.3	Methylquercetin isomer II	Flavonol
33.2	293.3	Dehydrodiferulic acid	Phenolic acid dimer (hydroxycinnamic acid derivative)
34.65	387.3	Medioresinol	Lignan
35.26	397.4	Sinapoylquinic acid	Phenolic acid derivative (hydroxycinnamic acid ester)

Table S2. List of identified xanthophylls along with UV-VIS and MS spectral data (molecule-related ion and fragments in positive ion mode)

N.	Carotenoid	λ_{max} UV-Vis (nm)	$[M+H]^+$	Fragments
1	Neochrome	400, 422, 450	601.5	583.4, 565.6
2	Luteoxanthin	399, 423, 449	601.5	583.4, 565.6
3	Auroxanthin	382, 402, 427	601.5	583.4, 565.6
4	Antheraxanthin	421, 441, 473	585.4	567.3, 549.5
5	Mutatoxanthin	405, 428, 453	585.4	567.3, 549.5
6	Lutein	422, 446, 472	569.6	551.5, 533.3, 476.4
7	all *E*-Zeaxanthin	426, 452, 478	569.6	551.5, 533.3, 476.4
8	9,9'*Z*-Zeaxanthin	338, 419, 446, 473	569.6	551.5, 533.3, 476.4
